# Schoenlein's Clinical Lectures in the Infirmary at Berlin

**Published:** 1846-01

**Authors:** 


Schoenlein's Klinische Vorträge in dem Charité-Krankenhause zu Berlin: Redigirt und herausgegeben von Dr. *L. Guterbock.* Zweite Auflage. 8vo. pp. 480. Berlin. 1842-4.

Schoenlein's Clinical Lectures in the Infirmary at Berlin: Edited and published by Dr. Guterbock. Second Edition.

In our last Number we gave a lengthened notice of the first half of this valuable series of clinical reports, affording our readers an ample opportunity of becoming acquainted with the opinions and practice of one of the most accomplished physicians upon the Continent. The subjects, to which attention was then more specially directed, were Typhus Fever, and Inflammation of the Lungs and Pleuræ. Those, which will now occupy us, are of a more varied character: and first of Acute Rheumatism.

Schoenlein very properly remarks that, in every case without exception of this malady, the state of the Heart and Lungs should be carefully examined; as, not unfrequently, there is an almost entire absence of all the *subjective* phenomena that are indicative of a lesion of these organs, and it is only by a diligent and repeated auscultatory exploration that we can arrive at any satisfactory knowledge. Hitherto, in such cases, the Heart has been more attended to than the Lungs; but the condition of both organs should be most narrowly watched; for there seems to be often as great a tendency to the supervention of Pneumonia or Bronchitis as of Carditis. Hence the importance of daily examining the respiratory sounds in the inter-scapular region, and of regularly ascertaining the state of the breathing in different attitudes and positions of the body. (Our readers will doubtless remember the observations of Dr. Latham upon this subject: *vide* the Number of this Review for July, 1845.) The matter of *perspiration* has usually a very peculiar acid smell in Acute Rheumatism; and we need scarcely say that the *urine* is always deep-coloured, and in most cases highly charged with the lithates of Ammonia and Soda. On this subject, we meet with the following remarks:

> "In rheumatism not only the urine, but other secretions also, as the perspiration, and even the matter of an abscess or ulcer, are found to contain an excess of acid matter. In one of Schoenlein's patients, the surface of an ulcer was observed to be covered with a layer of genuine crystalline matter, during the course of one night. We have good reason, therefore, to believe that lithic acid exists in the blood; for we know that it is present in much greater quantity than usual in the urine, and also in the matter of perspiration: it has also been found in the contents of rheumatic abscesses."

Now the knowledge of these facts is not only of theoretical, but also of direct practical, value; for, as long as the Perspiration has a distinct sour smell, and the Urine is abnormally acid, we may be assured that the morbid process, existing in the system, is not at an end. We cannot, therefore, urge too forcibly upon our readers the importance of attending to these phenomena. The disappearance of the local symptoms is often apt to mislead the medical attendant as well as the patient himself; and thus, perhaps, recourse may be had to the use of warm *baths*, whereby only a stronger impulse is given to the recrudescence of the disease. If there be a deficient elimination of the acid matter, present in the system, by the natural emunctory, the kidneys, the tendency of Endocarditis supervening, and of its occasioning deposits upon the cardiac valves, will be thereby much increased. Touching this point, it is worthy of notice that the absence or deficiency of the deposit in the urine, without being followed by cardiac lesion, is more frequently observed in women than in men.

The occurrence of a Miliary eruption on the *skin* in cases of acute Rheumatism is considered by our author to be, upon the whole, an unfavourable symptom. "I remember," says he, "that during one season, all the rheumatic cases were accompanied with this cutaneous complication, and that the cases so frequently proved fatal, that the eruption was regarded in the light of a plague"—a circumstance reminding one of the *febris sudatoria* that was so prevalent in the 15th and 16th centuries. The difficulty in the treatment of such cases consists in keeping the patient in an equable temperature, guarding him from cold on the one hand, and from excessive perspiration on the other.

As exerting a decided derivative action upon the bowels and kidneys (the best method of checking the threatened development of Miliaria), Schoenlein is in the habit of ordering a table-spoonful of the following mixture to be taken every two hours:—

℞. Infus. herb. Digitalis (℈ss) . . . ℥vj.

Nitri . . . . . . . . . . . . . ʒj.

Tinct. semin. Colchici* . . . . . . ʒij.

Syrupi Simpl. . . . . . . . . . ℥j. M.

In the *treatment* of acute Rheumatism, our author appears to trust chiefly to the use of direct antiphlogistic remedies, such as blood-letting, tartar-emetic, and so forth. We observe no mention made of the use of Mercury, even when cardiac or pulmonary inflammation has supervened. This, we consider, a great and serious omission in the practice of our Continental brother; although his opinion is shared, we know, by several modern writers in our own country.

When a Miliary eruption makes its appearance, Schoenlein recommends that the skin be bathed with an alkaline lotion. This, he considers, the best means to prevent the retrocession of the exanthem; and he greatly prefers its use, either to more active external stimulants applied to the skin, or to the internal exhibition of Camphor and Ammonia, as recommended by many authors. The alkaline wash has the advantage of not causing too much excitement of the skin, and yet enough to prevent the retrocession of the eruption; at the same time, it serves to neutralise the excess of acid matter that is secreted by the surface of the body. From a drachm to half an ounce of the caustic potash in a pint of water will be about the average strength of the lotion that should be used. It is to be applied tepid at first, and then gradually cooler and cooler. The irritability of the skin must regulate the strength of the solution to be employed; all that is required is, that it should produce a feeling of slight heat and pricking of the surface. The more profuse the perspiration is, the more frequently should the application be made. By the use of this wash, the eruption will speedily pass into the stage of exsiccation.

It is certainly very rare that genuine rheumatic inflammation ever terminates in the production of purulent matter. In cachectic states of the system, when the disease affects the synovial membranes of the joints, this termination has been observed to occur more frequently than under other circumstances. Whether the inflammation of the muscles in the following case was really and truly rheumatic, is doubtful.

Case 19. *Rheumatism of the Abdominal Muscles—Concentration of it in the Pyramidales—Discharge of Pus from the Rectum—Recovery.*

A robust plethoric man, 22 years of age, was seized, after exposure to cold, with a sharp pain over the *symphysis pubis;* it was much increased by pressure and motion: shortly afterwards, some degree of strangury came on. He was bled both generally and locally. Still the pain in the pubic region continued, although the urinary distress was relieved. The

---

* Schoenlein is of opinion that Colchicum (especially the tincture and wine of the seeds) is of marked efficacy in articular, but not in muscular, rheumatism. Its primary action, he says, is upon the intestines, and not, as Chelius supposes, upon the urinary organs. He has not found that it occasions the separation or excretion of the rheumatic sediment (in the urine, we presume—*Rev.*); nor does he consider that purgation is necessary to its curative action.

case was regarded as one of inflammation of the abdominal muscles (chiefly the *pyramidales*) with a tendency to its extending to the peritoneum: just as we not unfrequently observe that Pleuritis is preceded by a rheumatic affection of the thoracic muscles. When the *recti* or *pyramidales* are the seat of inflammation, it is apt to terminate in suppuration. In such cases, the pus is apt to have a somewhat urinous smell;—a circumstance which might occasion great alarm to the medical attendant, if he was not aware that it may occur without any lesion of the bladder; it is the result of the process of Exosmosis. (A similar remark has been made in cases of Abscesses situated near the rectum: the matter has sometimes a decidedly fæcal odour, although no direct communication exists between the gut and the abscess.)

Although the inflammatory symptoms were much mitigated by the means employed, there was a constant tendency to their recrudescence: from this circumstance, it was suspected that the case would terminate in suppuration. For some days, the patient was distressed with tenesmus; after straining one morning at stool, a quantity of purulent matter passed, with great relief to this symptom.* From this date, the patient's health rapidly recovered.

Case 20. *Rheumatism of the Abdominal Muscles—Peritonitis terminating in Exsudation—Removal of the Inflammation—Gradual Disappearance of the Swelling occasioned thereby.*

A woman, 28 years of age, was seized with sharp pains in the hypogastrium; at first they shifted about; but at length they became fixed in a spot equidistant between the ant. superior spine of the right ilium, the umbilicus, and the symphisis pubis. There was considerable tumefaction of this part; and a sense of fluctuation was perceptible in it. The patient had at first suffered from vomiting, and subsequently from a somewhat obstinate constipation. On examination *per vaginam*, no connection between the uterus and the swelling could be detected. The opinion, which M. Schoenlein formed of the case, was, that at first there was a rheumatic affection of the abdominal muscles; subsequently that the peritoneum, investing not only these muscles but also the intestines, had become inflamed; and that the result of this inflammation had been the effusion of purulent matter, probably between the abdominal and intestinal layers of the peritoneum. There was more than once a recrudescence of the pain in the swelling, accompanied with very considerable febrile re-action, nausea, &c.; the symptoms were always relieved by leeching, fomenta-

---

* Schoenlein remarks that, whenever, under such circumstances as the present case, the local suffering becomes much abated, while the febrile re-action continues but little abated, and assumes perhaps a remittent character—having distinct morning remissions and evening exacerbations—the physician ought to be on his guard as to the prognosis which he forms. In one case of rheumatic inflammation of the abdominal muscles the local symptoms entirely subsided, but the fever remained. In a few days, the catastrophe arrived: a quantity of pus was voided from the rectum and bladder. An abscess had formed in the pelvis, communicating with these two viscera.

tions, and so-forth. One day, a sense of tenesmus and dysuria was experienced; but no globules of pus could be detected in the urine with the aid of the microscope, although there was a considerable mucous sediment in it:—"it is no vain parade or charlatanary," our author here remarks, "to examine morbid products chemically and microscopically." The patient was at this time under the influence of mercury; a very considerable portion of mercurial ointment having been rubbed in around the seat of the abdominal swelling. As the feeling of tenesmus returned, it was thought advisable to ascertain the state of the *rectum* by a manual examination. Upon doing this, a distinct swelling was felt on the right side of the gut, intruding considerably into its cavity: the swelling was perceptible from the vagina also. It was deemed prudent not to make any incision into it, but rather to leave it to Nature, in the hope of its contents (if any) becoming absorbed.

After the patient had been in the hospital for upwards of a month, she left it almost quite convalescent. The abdominal tumour had disappeared, and that in the rectum was very much diminished. The rheumatic sediment in the urine, and the tendency to evening feverishness, had continued until just before the period when she went out.

In the *case* which is next reported, a violent Peritonitis occurred in a young woman, who had been suffering from acute rheumatism of the abdominal muscles, and had left the hospital before she was completely well. Notwithstanding the most active antiphlogistic remedies, the inflammation terminated in exsudation of lymph, and the effusion of a sero-purulent fluid. No trace of that crepitating sound, which Dr. Bright has said may be generally found by pressure upon a part where a plastic exsudation has taken place in the abdominal cavity, could be perceived; and, indeed, we cannot regard this (alleged) phenomenon as at all a trustworthy symptom of the lesion in question. The left iliac region was still considerably tumefied and tender, and it gave out a dull sound upon percussion. The patient was treated with *infus. digital.* and alkalis internally, and with mercurial ointment, to which some hydriodate of potash was added, externally. Things appeared to be going on somewhat more favourably than could have been suspected, when the vomiting and other unpleasant symptoms returned in consequence of an irregularity in diet, which the patient committed; the rejected matters were of a green or brown colour. At length the patient died on the 19th of June, four weeks after her admission into the hospital. On *dissection*, the abdominal viscera were found to be extensively agglutinated to each other by the exsudation partly of a fibrinous, and partly of a tuberculous, nature. The descending portion of the colon was found to have become ruptured, and to have given passage to the intestinal contents into the cavity of the pelvis.

We pass on to some illustrations of Puerperal Diseases.

In a case of violent puerperal Peritonitis, which was subdued by active antiphlogistic treatment, it was found, towards the decline of the inflammation, that the urine deposited a sediment of genuine purulent matter, as ascertained by chemical and microscopical examination. This phenomenon is not very rare,* according to our author. "I am far," he says, "from attributing this elimination of pus to the Digitalis which was prescribed yesterday; although I may remark that I have observed a similar phenomenon in cases of Empyema, after the exhibition of this medicine. The occurrence of this phenomenon is of the greater importance in a prognostic point of view, as it shews how far the inflammatory process has advanced, and in what stage it exists. * * * * * * * It is not uncommon in puerperal Peritonitis, that circumscribed purulent deposits form in the folds of the uterine ligaments, the ovaria, and Fallopian tubes: their existence can seldom be ascertained during the life of the patient. The appearance of pus in the urine is a fact of the utmost value in reference to our diagnosis: it is, as it were, a finger-post indicating the *stadium* of the inflammation."

Dr. Guterbock remarks, that it is still a difficult problem in Physiology, to determine in what manner purulent deposits are eliminated from the system along with the urinary secretion. We can scarcely imagine, he says, any other means of transit than the veins. If we admit, with some physiologists, that the globules of pus are too large to circulate with the blood through the capillary vessels, we must suppose that the purulent matter in its primary seat becomes partially dissolved, that it enters the blood in this thinner state, and is again separated from it in the kidneys as genuine pus. (*Vide* Müller's Physiology). The first assumption in this theory is of very questionable accuracy; as globules, of the size and form of pus-globules, have been repeatedly seen in the blood by various observers: these, Dr. G. is inclined to regard rather as epithelial cells of the vascular parietes than as lymph-globules, as some imagine. We must however, taking all circumstances into account, admit that it is by no means easy to account for the admission, in the first instance, of pus-globules into the blood, and for their subsequent elimination from it.

The 24th case is a very interesting example of Typhoid Puerperal fever, or *metritis septica,* as Boer calls it. The symptoms at first were very decidedly phlogistic, and required active depletion. As they subsided, the lochia became fœtid, and *phlegmasia alba* made its appearance in the right thigh. The occurrence of this malady clearly shewed that the venous system had become affected: no fewer than seven abscess-like swellings might be traced along the trajet of the femoral vein. At a later period, the basilic vein of the right arm, in which the patient had been bled, and one of the veins also of the left foot, exhibited symptoms of phlebitis. Purulent infection of the system soon manifested its presence by its characteristic shiverings, recurring at irregular intervals, and followed by flushes of heat, but without any distinct crisis. The patient too was now distressed with various pulmonic symptoms, which were, in all probability, connected with the formation of circumscribed abscesses in the tissue of the lungs.

---

* In a subsequently reported case, which also did well, the urine deposited a sediment, which was found on examination to contain purulent matter. It would seem, therefore, that pus may be eliminated from the system by the kidneys. The knowledge of this fact naturally suggests the importance of attending to the urinary secretion in all cases, where we have reason to suspect the occurrence of purulent infection of the constitution.

The following description of the morbid appearances in the veins of the uterus, &c., found on *dissection*, will be read with interest.

"The veins of the uterus and ovaria were, almost throughout their whole length, filled with pus. The inferior cava, near its transit by the liver, contained a puriform exsudation. In the entire right iliac vein there was a plastic tubular exsudation, and within this was found some gelatinous matter. The *trombus* extended as far as the middle of the crural vein, and from it into the smallest ramifications of the superficial vessels, the parietes of which had become so much thickened, that they resembled arteries more than veins. The veins of the right leg and foot were enlarged and thickened, but did not contain any lymph or pus within. The same appearances were observed in the right arm, and also in the veins of the abdominal parietes on the right side."

(The phenomena, constitutional as well as local, of these cases of purulent Phlebitis remind the reader, in some respects, of the history of Glanders, and such like septic diseases).

Schoenlein lays much stress upon the exact seat of the tenderness and pain in the hypogastrium, in enabling the physician to determine whether the case be one of Metritis or of Phlebitis uterina. If the pain be uniformly felt over one of the sides or lateral edges of the uterus, and not over its body or central part, we have reason to suspect that the veins are affected; as it is in the region indicated that the *plexus pampiniformis* is situated. The pain often extends from this spot to the side of the pubis and the labia, in the direction of the round ligament. When shiverings come on at the same time, there is good ground to apprehend that purulent Phlebitis has already commenced.

Uterine Phlebitis is rarely met with, except in the puerperal state. Schoenlein has, however, seen more than one case in which it occurred in young girls, in whom retention of the catamenia had taken place in consequence of an imperforate state of the vagina. He alludes to two cases where, after Textor had divided the obstructing membrane, symptoms of venous inflammation set in. This morbid condition is also apt to occur in cases of scirrhous, and other diseased, growths of the substance of the uterus: likewise when ill-conditioned ulcers or abscesses form about its cervix, or in the vagina; and, according to Boer's observations, when even slight ulcerations occur on the inner surface of the uterus, where the placenta had been attached. The prognosis is always very unfavourable, and especially if any shivering fits have set in. Schoenlein has never seen a case recover when these symptoms have occurred. The death will ensue in from two to twelve days after this event. The physician must never allow himself to form a favourable opinion from the mere circumstance of the subsidence of the hypogastric pain and tenderness; as long as the pulse remains rapid, and a feverish state of the system continues, he cannot (our author somewhat drolly remarks) with propriety sing *Te Deum*. We occasionally meet with, upon dissection, distinct purulent phlebitis of the uterine veins, although little or perhaps no distress had been experienced in the region of the womb during life—another argument to shew the necessity of a guarded prognosis.

The 26th case is one which exhibited the usual symptoms of Typhus gavior: it proved fatal. On dissection, no morbid appearance whatsoever was observed in the abdominal or cephalic viscera. There was however purulent Phlebitis in one of the veins of the right arm—the inflammation having commenced in, and spread from, the wound made in venæsection.

The 27th is a very interesting one; it is headed—

*Peritonitis—Attacks of Shivering—Inflammation of the Vena Portæ—Death—Dissection—Remarks on the Inflammation of the Portal Vein in general.*

The patient was a man 26 years of age. When admitted into the hospital, he was complaining of a violent pain in the epigastrium; it shifted its locality a little in different positions of the body. Pyrexia, diarrhœa, great thirst, and a high-coloured state of the urine present. The case was regarded as one of Perienteritis, and treated accordingly. On the third day, there was a well-marked shivering fit, followed by heat, but not by perspiration. Schoenlein says that he suspected the commencement of some mischief in the Portal system of veins. The rigors returned at irregular intervals; on some days there were several attacks. The treatment consisted in the inunction of mercurial ointment, the application of fomentations, and the internal use of calomel: the patient was speedily and rather severely salivated. Dr. S. assures us that, in several cases of lesion of the portal system, he has observed decided benefit from the use of saline baths (two pounds of common salt, and one ounce of muriate of lime being added to a usual bath); and he accordingly ordered them for this patient.* The icteric and febrile symptoms however continued to exist, and indeed gradually to become worse. The patient died in the course of about two months.

*Dissection.*—On opening the abdomen, an abscess was found about midway between the umbilicus and the point of the sternum (the seat of the pain during life); it was situated between the layers of the mesocolon. The abscess extended backwards in the direction of the Vena portæ. This vessel was found to be considerably enlarged, and, when laid open, to be filled with pus. The matter extended into its ramifications, the inner membrane of which was observed to be decidedly thickened. The parenchymatous substance of the liver was not affected.

In his comments upon this case, Dr. Schoenlein directs the attention of the reader to that aphorism of Hippocrates, wherein it is said—"Those, who suffer with violent fevers, rarely recover, if a shivering occurs on the sixth day." Our author regards the occurrence of this phenomenon, under such circumstances, to be a probable indication of the commence-

---

* Dr. Schoenlein says that he has found that the action of Mercury (and of Syphilis also) upon the system is much promoted and accelerated by the agency of muriatic salts, even in the form of saline baths. Hence it is that, in many places where the atmosphere is very saline, the employment of mercury in the treatment of the venereal disease is highly injurious. I observed this, he adds, at Venice, where the medical men are certainly very unsuccessful in the treatment of this disease with all mercurial preparations: indeed, any Venetian, anxious to get rid of the disease, should leave the place, and retire to the highlands of Lombardy. It has been remarked, that nowhere do we see so many people who have lost their noses, as in the city of the Lagunes.

ment of Phlebitis somewhere. As to the *symptoms of inflammation of the portal vein*, one of the most constant is (as far as we can well judge from our hitherto limited experience) a heavy oppressive pain about midway between the point of the ensiform cartilage and the umbilicus, and often, it may be, extending backwards in the direction of the spine: it is always increased by firm pressure. The usual signs of the biliary secretion being more or less imperfectly performed are generally present. Occasionally, blood is passed with the stools: indeed, Dr. S. is of opinion that the disease of Melæna is not unfrequently connected with the lesion, which we are now describing. The accompanying fever is usually violent—the *causus* of the ancient writers. Should the inflammation terminate in the formation of purulent matter, the fever will necessarily assume more of a typhoid, or perhaps of a hectic, character. If, in place of pus, plastic lymph is effused into the cavity of the inflamed vessels, the superficial abdominal veins are apt to become very considerably dilated, and the spleen also to be so much distended that, in the course perhaps of a few days, it may be felt outwardly to be excessively enlarged. When this is the case, we may expect to have all the usual symptoms of splenic disease; viz. tendency to syncope, confusion of sight, hæmorrhage from the left nostril, acid eructation or vomiting, and, subsequently, hæmorrhage from the bowels, which is almost always accompanied with extreme prostration. In one case, which has been described in Schmidt's Journal (1840), Schoenlein found, upon dissection, all the veins of the portal system almost quite obliterated.

Before quitting the subject of diseases of the Liver, we may introduce a few remarks on the—

### *Slowness of the Pulse in Icterus and Hepatitis.*

Schoenlein alludes to this circumstance on more than one occasion. In one of the cases of jaundice that are related, the number of beats did not exceed 30 in the minute. Frank has remarked that, even in active inflammation of the Liver, the pulse often does not exceed, and not unfrequently is below, the normal standard, although the other pyrexial symptoms are well marked; and he therefore cautions medical men not to allow themselves to be deceived by this occurrence, in their diagnosis of the disease. It is more than probable, that this diminution of the heart's activity is occasioned by the admixture of the pigment of the bile with the blood; for, we well know that, the absorption of this matter into the circulation has a marked sedative action on all the vital functions.

Our readers may like to know the treatment pursued by Schoenlein in jaundice. In one of the reports we find the following prescription.

Local bloodletting from the hepatic region—inunction of mercurial ointment upon the same part—purgative enemata—and this mixture, with the view of acting upon the bowels and kidneys at the same time

℞. Infus. herb. digital. (℈ss.) . . . ℥iv.

Tart. natronati . . . . . . . . . ℥j.

Mellag. graminis . . . . . . . . ℥j.

Aquæ laurocerasi . . . . . . . . ʒj. M.

A table-spoonful to be taken every two hours. Tamarind-whey to be used as ordinary drink.

Towards the decline of the disease, a mixture, composed of *decoct. rad. Graminis, extract. Taraxaci, and liquor kali acetici,* was ordered.

The object that is sought, by the administration of Digitalis in jaundice, appears to be the elimination of the colouring matter of the bile by the kidneys, the secretion from which is usually much increased by this medicine. It has been said by some writers that the pulse is apt to rise under the use of Digitalis in this disease—a circumstance which, if really true, may be accounted for by supposing that, in proportion as the bile is separated by the urine from the blood, so will the action of the heart be relieved from the *incubus* that rests upon it.

It is always judicious practice to keep up free evacuations from the bowels and kidneys, whenever the biliary excretion is interrupted; as the chief danger of such obstruction is the rapid supervention of alarming cephalic symptoms.

In *Colica Pictonum*, the constipation of the bowels is doubtless owing to the same cause, as the paralytic weakness of the muscles of the extremities, that is so frequently met with after poisoning from the salts, not only of Lead, but also of Copper and Arsenic. There occurs, under such circumstances—we have every reason to believe—a lesion not only of the irritability, but also of the very substance, of the muscular texture; and indeed upon this organic change, medical jurists have, of late years, laid considerable stress in cases of poisoning from the last-named metal. In cases of Lead Colic, the longitudinal fibres of the Colon have been observed to have undergone a very perceptible change. In the present instance (one of inflammatory *Ileus* in a person who had previously suffered from lead colic), there is most probably not a short and circular, but a wide or diffused, constriction of the gut. The patient was bled and leeched, and an injection with castor-oil was administered. Scarcely any thing was administered by the mouth, except a few drops of a solution of Morphia, to check the vomiting. The severe symptoms were relieved for a short time, but a fatal collapse speedily supervened. On *dissection*, the transverse Colon was found to be completely obstructed, in consequence of an angular twist, which entirely prevented the passage of its contents downwards. The parts exhibited the marks of an old adhesive inflammation. The longitudinal fibres of the gut were observed to be atrophied, and to have lost all their colour. The ascending Colon was distended with gaseous and fæcal matters.

"This case affords us an opportunity to compare its phenomena with those of some similar ones that have been related by Dr. Buchanan of Glasgow, in the 24th volume of the London Medical Gazette, but which differ from ours in the circumstance, that no marks of inflammation or of fibrinous exsudation were observed in any recorded by that gentleman. Such dislocations of the colon are always the consequences of the elongation of this gut; they have been described by several Italian as well as English physicians. Besides the angular or elbow-shaped flexure, Dr. Buchanan has represented, in the sketches which accompany his memoir, a twisting round of the gut upon its axis. These cases should be characterised more particularly by the great tympanitic distension of the abdomen, which is thereby sometimes as much enlarged as in cases of Ascites: the bowels too are usually much confined, while no obstruction can be discovered in the rectum. In consequence of the diaphragm being pushed up into the cavity of the thorax by the enormously distended bowels, the breathing is always much embarassed."

In the treatment of such cases, Schoenlein very wisely remarks that the well-timed use of antiphlogistic remedies, and more especially of local or even general blood-letting, will often be more serviceable than that of any other remedies. The experience of our *late*—how sad the associations which this little word suggests!—most valued friend and counsellor, Dr. James Johnson, was quite in accordance with this precept. In the number of this Journal for January 1845, p. 72, he has remarked:

"In the course of a long and practical life, we have met with many cases of the above kind (invincible constipation)—some fatal, some fortunate. For many years past, we have made up our minds to this point, that, whenever constipation exceeds two or three days, inflammation and its various consequences, adhesions, ulceration, perforation, &c. are to be dreaded, and are the grand objects of prevention."

The *accumulation of fæculent matters* in some parts of the intestinal canal is apt to be mistaken for scirrhous and other tumours of the viscera. I once saw a man, says our author, whose case had been declared by an experienced physician to be one of scirrhous swelling in the abdomen,—in consequence of a hard immoveable lump that was situated at the side of the umbilicus—but who fortunately was speedily relieved of all his inconvenience by the use of warm-water enemata and castor-oil. He subsequently died of another disease; and, on dissection, it was found that the ascending Colon was contracted at one point, and that the gut, nearer the Cæcum, was much distended, and quite filled with hardened fæcal matters. In this case, the swelling was not much bigger than a man's fist; but, in another, it was as large as a child's head, and the fæces were as hard as cannon-balls.—It has been frequently observed that the fæces are apt to accumulate in the cæcum; and it is well for the medical practitioner to be aware of the fact that, in such cases, there may be no constipation; but that even a diarrhœa may be present. A passage is then formed either through the substance of the hardened fæces, or between them and the walls of the gut; and along this passage the thinner matters may flow on, while the thicker and more consistent are retained. A case, therefore, of seeming diarrhœa may actually be one of inveterate lodgement. I recently observed something of this sort in the case of a young female, who was recovering from Peri-enteritis of the small intestines, and inflammation of the mucous membrane of the colon. The stools had been frequent, thin, and mucous for several days; but then a large quantity of solid scybalous fæces were evacuated: no sooner did this take place than the diarrhœa entirely subsided. This circumstance is not unfrequently alluded to by the older writers, in their descriptions of Dysentery. The bowels, above the seat of the accumulation, are usually much distended with flatus. The circumstances now mentioned must satisfy every reader of the necessity of a physician carefully examining for himself the condition of the alvine evacuations, and of not trusting to the mere report of patients, in cases of obstinate Diarrhœa. It will be prudent, too, sometimes to ascertain the state of the rectum by a manual examination.

### *Cancerous Disease of the Stomach.*

In a case of this formidable malady, recorded in these Reports, the diagnosis was very materially assisted by the microscopical examination of the matters that were rejected by vomiting. Cellules, having all the characters of those which are usually considered to be characteristic of cancerous formation, were found mixed with the food. Along with these cellules, were numerous fatty globules, a few blood-corpuscles, and a good many epithelial scales. The treatment pursued was of the simplest description. Schoenlein very properly condemns the use of active medicines, such as Iodine, Bromine, Mercury, and so forth, in diseases that are universally acknowledged to be beyond the reach of art. Physicians, he remarks, seem often to forget that Medicine has its limits, and that all attempts to transgress these are usually punished with a decided aggravation of the existing distress.

The abdomen of the patient was fomented with a decoction of Belladonna; and an Emulsion, containing Castor-oil and Cherry-laurel water, was ordered for internal use. An occasional warm bath, and a mild Enema, as circumstances might require, were also prescribed. Subsequently, small doses of Morphia were administered, with the view of procuring sleep. Under the use of these gentle remedies, the sufferings of the patient were very much relieved.

The following general remarks on this awful malady may be aptly introduced here.

"If the seat of the disease be at a considerable distance from the pylorus, the symptoms are usually much milder than under the contrary circumstances; the vomiting also will be less severe. But even when the pylorus itself is the part chiefly affected, we occasionally find that, in the advanced stages of the disease, when the diameter of this orifice has become enlarged by the progress of the ulceration, the vomiting and constipation either cease altogether or are very materially abated, and the patient is relieved from some of the most distressing sufferings to which he had been previously subject. * * * * I have seen, says our author, cases of morbid degeneration of the stomach where, although the ulcerative process had been fairly established for a length of time, all the symptoms, indicative of gastric disease, subsided so entirely that the patient has eaten his food with a perfect appetite and without suffering any subsequent inconvenience. This subsidence of the morbid phenomena has been observed to occur more especially in cases, where pulmonary phthisis had supervened (as not unfrequently happens) in the course of the gastric malady. When this complication exists, it will be found that, just in proportion as the Lungs become more seriously affected, the gastric distress diminishes in severity. The difficulty of the diagnosis may then be increased the more, by the epigastric tumour becoming less distinctly perceptible than it had been before; for the diseased pylorus is apt to be drawn further back towards the spine, and become firmly adherent to the concave surface of the liver, by which it is in a great measure covered and concealed. The microscopic examination of the egesta from the stomach may, under such circumstances, be the only decidedly satisfactory symptom of the existing disease that we possess: hence the very great importance of this mode of investigation. The medical man must, therefore, be on his guard not to let himself be deceived by these fair but false appearances. Occasionally, the biliary secretion is very greatly diminished in quantity; and, as no proper digestion can take place without the aid of the bile, it will sometimes happen that the food is carried rapidly through the bowels, and voided (having undergone but little change) within an hour or so after it has been swallowed. This circumstance will account for the frequent craving for food that may be experienced. This remark applies to various diseases of the Liver, in which the secretion of the Bile is more or less deficient, provided the bowels are at the same time in an irritable condition. As a matter of course, if this latter condition be not present, the absence of the bile is usually attended with decided constipation."

After relating the particulars of a case which is headed—*Ascites—hypertrophy of the Spleen—tubercles in the Lungs—pus in the Urine—scrofulous phthisis of the Kidney—paracentesis of the Abdomen—urea present in the dropsical fluid—death*, and in which, on dissection, the left kidney had become converted into a mass of soft caseous matter, our author makes the following observations on "Scrofulous Degeneration of the Kidneys."

"The symptoms in the early stage of the disease have often a great resemblance or analogy, in the mode of their development, to those which usually indicate the existence of tubercles in the lungs. The constitution exhibits the marks of a scrofulous temperament, and the patient—not unfrequently a boy or girl of from seven to ten years of age—is always more or less ailing, especially after exposure to cold, or any irregularity of diet. Complaint is made of an uneasy feeling in the loins; and, when a more particular enquiry is made, it is discovered that the uneasiness is felt not in the course of the spine, but rather on one side of it. There are frequent calls to pass the urine, especially when the weather is cold and chilly. This frequent desire is often greatest during the night (just as we observe to be the case with the cough in tubercles of the lungs), and the child may therefore be apt to wet his bed during sleep. The urine at first is observed to be pale and clear, or partially opaque and troubled from an admixture of mucus; this comes from the bladder; and it is well known that, in the early stage of pulmonary phthisis, the sputa are at first from the trachea. As the disease advances, a more decided pain is experienced in the renal region, and then the urine may exhibit marks of an admixture of blood with it: here again we have an analogy to the hæmoptysis so frequent in the second stage of pulmonary tubercles. If the patient be arrived at the age of puberty, there will perhaps be erections, and crampy pains in the scrotum at the same time. Hitherto the renal affection may be amenable to medical treatment. With respect to the treatment, local blood-letting, the ioduret of Potassium, or of Mercury (rubbed in upon the loins), the use of salt baths, and of a mild unirritating diet—these are the principal means to be employed. When the disease is further advanced, we can only relieve symptoms."

The 39th Case is an interesting one of *Diabetes Mellitus* associated—the connection is by no means unfrequent—with *pulmonary tubercles*. The influence of opium, in diminishing the urinary secretion, was well marked and most beneficial: a grain was given twice a day. It produced no drowsiness, nor any constipation of the bowels; and indeed, as Schoenlein remarks, it seldom exhibits either of these effects in cases of diabetes. From this circumstance, we see with what little confidence we can sometimes predict the effects of various remedies, in certain morbid states of the system. With respect to the presence of sugar in the urine, "I am of opinion," says our author, "that, like that of albumen in this secretion, this morbid admixture may be owing to various circumstances. I have found albumen in the urine in the progress of very different diseases; for example, in acute Rheumatism, in Ague, in some cases of Typhus fever, and in Scarlatina. So I believe it is with the case of saccharine matter in this fluid; and, if so, then surely we must give up hunting after any specific remedy for the malady, and rather seek to discover its cause, or at least the morbid condition of the system with which it is associated, and apply our therapeutic resources accordingly. If it occurs in connection with scrofulous disease, we should try the effects of the hydrosulphuret of Ammonia, various preparations of Iodine, a nutritious regimen, and so forth. But diabetes may exist quite independently of this diathesis, and may then require a very different course of treatment. Nothing is more injurious to the credit of the healing art than the too common practice of prescribing for the name, and not for the symptoms, of a disease."

Schoenlein again alludes to the necessity of examining the urine at different periods of the day, if we wish to arrive at accurate results as to its true condition. "In cases of spleen-disease," he remarks, "I have frequently observed that there was always a most copious deposit of the lithates, having a peculiar purplish colour, after any meal; while at other times of the day there was little or none to be discovered. In such circumstances, the patient will continue to lose flesh, although his appetite may remain good all the while. A similar fluctuation in the state of the urine will be found in Diabetes, Albuminuria, Suppuration of the Kidneys, &c. Again, in cases of Scarlatina, towards the decline of the disease, the urine will sometimes be found quite normal in the morning, while it is more or less decidedly albuminous in the evening.

*State of the Urinary Organs in Scarlatina.*

There is a peculiar phenomenon, that occurs in the convalescent stage of this Exanthem, which is interesting and worthy of notice in many points of view. That there is an exfoliation of the mucous membrane of the mouth and fauces, is known to every medical man; but few are aware that a similar process not unfrequently takes place along the whole course of the urinary organs. That such is the case may be discovered by examining the urine with the microscope. If this be done, we shall often find an innumerable number of epithelial scales, which to the unassisted eye look, in the mass, like a mucous sediment or opalescent muddiness. Schoenlein is of opinion that this exfoliation of the mucous membrane of the uropoietic organs is the real cause, that predisposes the patient to that form of dropsy which is so apt to occur after Scarlatina, and in which the urine is well known to frequently contain a number of blood-globules, as well as a quantity of albumen. Such a condition of the urinary secretion may very reasonably be regarded, as indicating a state of high irritation of the mucous surface along which it flows. It is, therefore, a very natural and obvious deduction that a patient should never be pronounced quite convalescent by his physician, until not only the cutaneous desquamation has entirely ceased, but the urinary secretion also has resumed its healthy condition in every respect. If this rule were more uniformly followed in practice, many of the most unpleasant *sequelæ* of Scarlatina might unquestionably be avoided. The patient should be strictly guarded from cold, and the state of the urine be sedulously watched for several weeks after the decline of the eruption.

The 41st Case is one of *Erysipelas* of the face, which occurred in a middle-aged labouring man, who had been much addicted to excessive drinking. The cutaneous eruption was very imperfectly developed, and there was much cerebral irritation present. It was not deemed wise to administer any opiate with the view of assuaging the head-symptoms, although they partook largely of the characters of *Delirium tremens*. The wiser course appeared to be to induce a more decided derivation of the blood to the surface, in the hope of exciting the torpid exanthem to greater activity, and at the same time to stimulate the bowels and kidneys by appropriate remedies.

To attain these objects, 20 leeches were, first of all, applied behind the ears. The patient was afterwards placed in a large tub, and a quantity of cold water poured over him. The following mixture was ordered for internal use:

℞. Infusi herb. Digitalis (℈ss.) ℥iv.

Nitri ʒij.

Tart. stibiat. gr. j.

Mucilag. gummi Mim. ℥j.

Syrupi simpl. ℥j. M.

A table-spoonful to be given every hour.

The patient ultimately recovered. The following remarks, on *the use of the cold affusion in Erysipelas*, are deserving of especial notice.

"In the case of this patient,—whose condition upon admission was most unpromising, in consequence not only of the livid aspect of the Erysipelatous eruption, but also of the tendency to the supervention of Delirium tremens—we had recourse to a remedy whose use is not without danger, but which the circumstances of the malady seemed to require. The cold affusion is not to be employed in all cases, nor in any without great consideration; for very serious consequences may result from its injudicious adoption. According to my experience, it is when the exanthem exhibits a livid or bluish colour; when the affected skin is little swollen, but is dry and is the seat of a burning prickling heat; when the temperature of the body is unequal, the extremities being perhaps cold, while the head is hot; and when the accompanying fever is of a nervous typhoid type, that the cold affusion is sometimes of great service; indeed, the only remedy which holds out any prospect of decided advantage. When this is the case, we observe, after its employment, that the skin becomes more turgescent, that the colour of the eruption is more decidedly red than it was before, that the delirium abates, and the tongue is moister—changes, which indicate that the remedy need not be repeated. If, however, the former state of things returns, it will be necessary again to have recouse to the affusion. I have seen another form of Erysipelas, in which the cold affusion has been employed with signal advantage: this form prevailed epidemically in the hospital at Zurich in the year 1836. The exanthem did not come out upon the surface fully or regularly; there were only patches scattered here and there; and these appeared for a short while, and then vanished. At the same time, the sensorium was much disturbed. A few of the patients lay in a state of stupor; but in most there was a strange confusion of mind, so that they left their beds, and went about the wards, as if they were going to their ordinary work. Some of them squatted themselves down upon the floor, fancying that they were on the night-stool, or in the water-closet. In most instances, the pulse was but little excited, except towards evening; in some, it was below the natural standard.* After the use of the cold affusion, the ex-

* An unusually slow pulse is a common phenomenon in many cerebral diseases. Indeed, the presence of this symptom in some cases, where the diagnosis is difficult and ambiguous, may serve to assist the medical man in the judgment which he ought to form of the nature of his patient's malady, anthem came out much more freely, the face became redder and more swollen, and altogether there was a more distinct vascular action than had existed before. In most of the patients, the effect of the douche was of short duration; it required to be repeated in the course of a few hours, and even a third time. There is still another form of the disease in which the remedy has been used with marked benefit. As we saw in our present patient that the erysipelatous affection stood still, and that the skin, under the exfoliating integument, exhibited a new redness, so we not unfrequently have occasion to observe a similar phenomenon in elderly persons, who are affected with a constant erysipelas of the face; the red patches upon the point of the nose or over the zygomatic arch, for example, being surrounded with a red ring, and presenting a shining surface. If in this state the head becomes affected, and if the patient is delirious, has a rapid weak pulse, a dry coated tongue, and the stomach is perhaps irritable to vomiting, I believe that there is no remedy that promises so much benefit as the judicious employment of the cold affusion. The old physicians in such cases recommended an emetic in the first instance, and afterwards the use of camphor and such-like stimulants; but I greatly prefer the above treatment."

There is a tendency to Dropsical complaints occurring after some forms of Erysipelas, as after Scarlatina. Not unfrequently, too, we find that erysipelas of the face leaves behind it a swelling of the submaxillary or cervical glands, or an inflammation of the internal ear—two very common results of scarlet fever. Mania, as well as other cerebral affections, are also occasional consequences of erysipelas of this part.

### *Influence of Menstruation upon the Exanthemata.*

The appearance of the Catamenia, in the course of any of the acute exanthemata, is always to be regarded as an unpleasant symptom. Cases, indeed, occur, where the act of menstruation seems to have a truly critical salutary influence upon the existing fever; the violent pyrexial and the grave nervous symptoms subsiding immediately upon its development. But the very opposite state of things is much more common; and most experienced physicians must have met with cases where the occurrence of this secretion, during the eruptive stage of Measles for example, has been speedily followed by alarming oppression and anxiety, and perhaps by death. If the case does not prove fatal, the eruption will generally be observed to become more or less livid, and to have petechial spots mixed with it; these spots may be distinguished from the proper exanthem, by not disappearing under the pressure of the finger. A not unfrequent symptom in such circumstances—and one of the worst augury—is a very fetid odour of the breath, the tongue being at the same time parched, and covered with a dry brown crust. Again, when the Catamenia are entirely suppressed, the prognosis must be extremely unfavourable. It is therefore obvious that the state of this function must influence very materially our opinion, as to the danger of exanthematous fevers in menstruating females. Many of the older writers have alluded to the very unfavourable change that is apt to occur in the eruption of small-pox, by the occurrence of menstruation. The pustules are apt to become livid from the admixture of a sanguinolent fluid with their contents, and large patches of ecchymosis to make their appearance on different parts of the surface. The danger is the greater, if the catamenia occur upon the seventh or eleventh day of the exanthem.* An inexpressible oppression or anxiety is then apt to come over the patient; she feels inwardly assured (a genuine *clairvoyance*) of her approaching dissolution; her pulse becomes exceedingly weak and rapid; her feet are cold; and in a few hours, perhaps, death will close the scene, although everything had, shortly before, seemed propitious and favourable.

Dissection in such cases usually discovers an unusual fluidity of the blood, and—a common consequence of this morbid state—a dark-red colouring of the inner surface of the bloodvessels; such as we frequently observe in cases of death from lightning. This appearance has been erroneously attributed to inflammation; whereas it is the mere result of imbibition or Endosmosis of the vascular parietes.

*The Intermittent Character of certain Diseases of the Nervous System.*

The tendency to intermittence of the symptoms, in various lesions of the Cerebro-spinal System, is a circumstance that ought to be well known to medical practitioners; as they might readily fall into a serious error in their diagnosis, if they were not aware of it. In the acute Hydrocephalus of children, the symptoms in the early stage of the disease very usually exhibit more or less of intermittence. I have repeatedly noticed this circumstance in the genuine disease—not to mention that form of fever which has been called *intermittens maligna cerebralis,* and which has been so often mistaken for it. In the after-part of the day, the symptoms of cerebral irritation usually come on, and continue till about midnight; then they gradually subside, and the child appears to be almost quite well, so that its parents may probably suppose that it was nothing but a casual illness. Again, however, in the afternoon, the child becomes feverish and uneasy; and the same aggravation and subsidence of the symptoms occur as on the preceding day. This state of things will, if unchecked, continue until the pyrexia and head-distress become permanent. When such is the case, mischief has already fairly commenced, and will perhaps utterly defy the best directed efforts of the most experienced physician. In inflammatory affections of the Spinal Marrow also, this intermittence of symptoms is not unfrequently observed in the early stages of the morbid action.

*Issues on the Scalp in Chronic Cerebral Diseases.*

The establishment of a caustic drain on the scalp over the suspected seat of the lesion, in cases of chronic cerebral disease, has proved in my hands a remedy of very decided utility. I remember the case of a woman who suffered the most agonising pain, notwithstanding the use of the most powerful narcotics, in consequence of a carious destruction of the petrous portion of the left temporal bone: the patient was much emaciated, and was affected with hectic fever. A potential cautery was applied over the squamous plate of the bone. As soon as the cauterised skin began to discharge, the relief was most gratifying; and the progress of the fatal event

---

* In a case of Scarlatina, related in these reports, Schoenlein advises medical men never to give a confident favourable prognosis in this disease, until the eleventh day is past.

was very materially retarded. Women, who have, at some former period of life, suffered from a chronic irritation of the cerebral meninges, are apt to be affected, as life advances, with a most severe pain on the crown of the head, either along the course of the Sagittal Suture or a little to one side (usually the right) of it. When such a case proves fatal, the cerebral meninges are generally found to be thickened and agglutinated together, and the glandulæ Pacchioni to be enlarged; this increase of size being sometimes so great as to have caused a considerable absorption of the bone over them. In such cases, all opiates and antispasmodics are utterly useless, and perhaps decidedly injurious: the only safe and judicious remedies are repeated local bleedings, and the establishment of an issue over the seat of the lesion.

For exciting the irritability of paralysed muscles, the most important of all remedies is unquestionably *Electro-Magnetism.* I have seen many remarkable cures effected by its use. Not a few cases of paralysis of the generative organs have been speedily and effectually benefited by it. *Strychnine*, also, will be found to have very remarkable powers in curing certain forms of paralytic weakness. Its employment requires much discrimination and judgment; for, if administered too early, before the cerebral lesion has been very materially modified, mischief, and not benefit, will be the result. The same remark is applicable to the use of electricity.

Before drawing our notice of this truly valuable volume to a close, there are two or three incidental topics which attracted our notice during its perusal, and which therefore may not be undeserving of the reader's attention.

### *The Doctrine of Post-scabial Diseases.*

"Of late years the recognition of post-scabial diseases, an ancient dogma in medicine, has been not only abandoned, but ridiculed and despised. In 1807, Autenrieth wrote an admirable memoir on the subject; it was by him, and not by Hahnemann, as is generally supposed, that public attention has been drawn to the subject in the course of the present century. The discovery of the scabial Insect has brought the whole matter once more into dispute. That this animalcule is often present in cases of Scabies, I can testify myself, as I have seen it on more than one occasion; but that its presence upsets the old doctrine of post-scabial disease, I must distinctly deny. I will not insist upon the direct experiments and observations in olden times; nor upon the fact that, when, after the diappearance of the itch, another disease sprung up, the latter either stood still, or entirely ceased, as soon as the former re-appeared. I will take another line of argument, and enquire of the opponents of the doctrine, How is the itch developed? At first, we observe minute papules; these are speedily changed into vesicles and pustules. Now it has not been shewn that, on the earliest appearance of the papules, any trace of an insect is to be found: this would be a *filius ante patrem.* Here, therefore, there is an obvious contradiction.—Moreover, it is admitted by Raspail, and all his followers, that the insect is far from being present in every vesicle of the Itch; and yet, if the animalcule be the cause of the disease, it ought surely to be so.—There is another fact that deserves notice; and it is this. The insect is found only in the disease of recent standing, and not in old and chronic cases.—The objection, that the disease may be communicated by the inoculation of the insect, cannot have much weight; for the insect could not be well conveyed from the vesicle, without carrying along with it some of the contagious matter. To render the experiment convincing, the microscopic insect would require to be well washed and cleansed with a brush! for we all know what a minute quantity of contagious matter will suffice for the purposes of contagion.—The question is, therefore, by no means definitively settled by the discovery of the Itch-animalcule, as many writers have supposed. I will frankly confess that I have no doubt myself as to the existence of post-scabial diseases. It has been frequently observed (especially in old persons) that a peculiar ulceration of the skin, more particularly around the joints of the lower extremities, is apt to occur after the disappearance of a scabious eruption; and that, if this ulceration (the *ulcus psoricum* of certain authors) be suddenly dried up, internal diseases of a decidedly peculiar description are apt to arise. As I have already said, there is no doubt in my own mind that Scabies is apt to occasion various consecutive maladies, and among these I may mention diseases of the Heart."

### *Memoranda on Rheumatism.*

It is one of the most difficult problems in practical medicine, to determine the exact degree in which we should encourage the elimination of the diseased product (the *materies morbi*) of Rheumatism from the different emunctories of the system. We know that, if we act too powerfully upon the skin, this tissue becomes, so to speak, saturated with the acrimonious matter *(acre rheumaticum)*; and some cutaneous affection—as Erythema or Miliaria—is apt to be induced. In the same manner, if we direct the action exclusively upon the kidneys, certain morbid changes in the functions of these important excretory organs may occur in consequence. Thus, Nephritis has been known to take place in some instances; while, in others, the urine has been found to become albuminous, or to contain such an excess of lithic acid, as to give rise to renal calculus. A morbid material or product is unquestionably present in this disease, and must be eliminated from the system before we can expect to obtain a cure. There are three channels or emunctories by which this process of elimination may be effected—the skin, the kidneys, and the bowels.

"It is a curious and somewhat puzzling circumstance in the history of Rheumatic diseases that, in many cases, the constitutional symptoms, without any discoverable cause, exhibit a marked nervous character, while the local ones are unattended with any material or physical alterations of the parts affected—in other words, there is no formation of any morbid production. In such cases, we have always to fear the supervention of some internal mischief. Sometimes the muscles, that are the seat of the chief pain, become affected with Paralytic weakness; at other times, I have seen the cerebro-spinal axis struck with a genuine *nervous* Apoplexy. This is a very dangerous form of disease; and, should the attack prove fatal, no decided morbid appearances are almost ever discoverable in the encephalon. It has been the fashion of late years to deny the existence of this form of apoplectic seizure; it is, however, not unfrequently alluded to in the writings of the older physicians. That there is such a disease, I feel quite convinced; although I readily admit that it is by no means easy to explain many of its phenomena. I saw a well-marked case of it recently, in the person of a young woman, who had been seized with a paralytic weakness of the lower extremities. She speedily recovered the use of them by the employment of alkaline baths, and the exhibition of *ammonium pyro-oleosum.* She returned to her work as kitchen-servant too soon. A few days subsequently, she was brought back to the hospital in a state of apoplexy: the attack proved fatal. No morbid appearance was discoverable upon dissection in any part of the brain. Such cases are by no means unfrequent. It is worthy of notice that they stand in an inverted relation, in point of frequency, to the *materiality* of the rheumatic process. The greater the amount of the formation and excretion of the acid matters from the system, the less tendency will there be to this formidable malady. These torpid forms of nervous disease belong to the worst kinds of articular Rheumatism."

*The Therapeutic Action of certain Medicines.*

"We often observe that a medicine reaches a point of saturation, so to speak, in its effects upon certain organs, so that no increase of effect is obtained by an increase of the dose. It would be instructive to ascertain whether this operation of a remedy is at all correspondent with the induction of other positive and obvious phenomena; for example, whether the effects of Digitalis as a diuretic are at all influenced by the amount or degree of narcotism, which it may happen to induce. If such were the case, we should have a guide or indication for the discontinuance of the medicine. The older physicians were well acquainted with the circumstance alluded to. According to them, so long as a remedy corresponded with, or was suitable to, the malady for which it was prescribed, its injurious or poisonous effects are not apparent. Thus, enormous doses of Nitre may be given in inflammatory disorders, without causing any disturbance of the digestive organs; its operation and action are entirely expended upon the morbid process: when this is over, then indeed will be experienced its unpleasant effects, especially if the medicine be continued in the same doses as before. A similar remark has been made respecting the effects of Mercury upon the system. Now there is a good deal of truth in these statements. A physician must have often occasion to observe that the diuresis, caused by the operation of a diuretic, will suddenly become very much diminished, although the medicine be continued in the same doses. If, however, the use of the medicine be suspended for even a single day, it will be found that, when resumed, it is as potent as ever it was; although there has been no increase in the quantity administered. In such cases, we shall often find that the success of the treatment depends not upon the degree of irritation produced on any one organ, but upon the adaptation of the remedies to the peculiarities of each individual case. It is far from being necessary to have frequent recourse, in the treatment of disease, to the more potent or very active remedies; the judicious use of the more gentle will often effect the desired object, with much less suffering to the patient, and less injury to his constitution."

As an illustration of these remarks, let us hear what Schoenlein says of Broussaiism.

"At the time when the antiphlogistic method of treatment was in full swing, and the whole of practical medicine was comprised, so to speak, within a nutshell, mere frequency of the pulse was almost always set down as a symptom of fever; and wherever there was fever, then there must be inflammation; and where there was inflammation, blood must be drawn more or less freely. When a patient died, the question invariably was, has he been bled?—and how often?—and, if it was answered ten times, the immediate reply was—what a pity that he was not bled the eleventh time! I have myself seen chlorotic girls bled, merely because there was palpitation of the heart, and the pulse was unusually rapid. As a matter of course, many of the unfortunate victims sunk under the treatment. I well remember the case of a medical dignitary, who was treated strictly according to the most approved method of the antiphlogistic plan for some fancied phlogistic disease, and in whose body the appearances found on dissection clearly indicated not inflammation, but positive anæmia."

What Sydenham has said of the injudicious treatment of Gout, may be applied with still greater force and truth to that of Fever, under the blood-letting mania which so long prevailed among the continental physicians—*fidenter assero, multâ et longâ observatione suffultus, maximam partem eorum, qui Podagrâ periisse putantur, non tam ipso morbo quam indebitâ medicatione fuisse peremptos.*

We cannot pay a higher compliment to Schoenlein than to say, that the present volume is deeply imbued with the spirit of the writings of our great countryman.

